# Quality Assessment of Box Materials for Long‐Term Archival Storage: VOC Emissions Are Not a Significant Concern

**DOI:** 10.1002/cplu.202500337

**Published:** 2025-12-13

**Authors:** Randa Deraz, Fabiana Di Gianvincenzo, Katharina Schuhmann, Manfred Anders, Jasna Malešič, Irena Kralj Cigić, Abdelrazek Elnaggar, Matija Strlič

**Affiliations:** ^1^ Heritage Science Lab Ljubljana Faculty of Chemistry and Chemical Technology University of Ljubljana Ljubljana Slovenia; ^2^ Van 't Hoff Institute for Molecular Sciences University of Amsterdam Amsterdam Netherlands; ^3^ Research and Development Department ZFB ZENTRUM FÜR BUCHERHALTUNG GmbH Leipzig Germany; ^4^ Research Department, National and University Library Ljubljana Slovenia; ^5^ Archaeological Science and Excavation Department Faculty of Archaeology Ain Shams University Abbasia Egypt; ^6^ UCL Institute for Sustainable Heritage University College London London UK

**Keywords:** acetic and formic acid, archival storage, paper degradation, risk assessment, volatile organic compounds (VOCs)

## Abstract

A potential impact of archival storage materials that seems to be of increased concern is the volatile organic compounds (VOCs) emitted from such materials. In this study, VOC emissions from cardboard and polypropylene were analyzed using thermal desorption gas chromatography–mass spectrometry (GC–MS) and ion chromatography (IC), with particular attention given to acetic and formic acids, and their impact was evaluated using Oddy tests. While the latter revealed that some nonarchival grade packaging materials could represent a risk to both metal and paper, which can be explained by VOC emissions measured using GC–MS, acid emissions measured at room temperature provided a different picture. Equilibrium acid concentration was modeled in archival boxes, which turned out to be insignificant in comparison with the current standards for archival air quality. This suggests that even nonarchival quality boxes do not significantly contribute to the degradation of paper, which emits its own VOCs, including organic acids. With suitable air exchange rates, the concern about box materials significantly contributing to VOC‐induced degradation of paper stored within is thus not justified. Additionally, Oddy tests and other emission tests at elevated temperatures need to be re‐evaluated in relation to their value to preventive conservation of organic materials.

## Graphical abstract

The image summarizes the main finding of our assessment of VOC emissions from archival box materials. VOCs from archive boxes were analyzed using GC–MS, IC, and Oddy tests. While some storage materials showed hazards at elevated temperatures, acetic and formic acid emissions at room temperature were very low. Modeled equilibrium acid levels inside boxes were well below the 100 ppb threshold (PAS 198:2012), indicating that the stored paper—not the box materials—is the primary source of VOCs. Even with minimum air exchange, the box materials do not contribute significantly to paper degradation caused by VOCs.

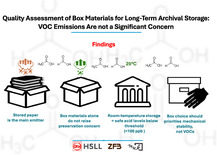

## 1 Introduction

The long‐term storage of historic paper‐based collections is an ongoing challenge faced by decision makers in GLAM institutions (galleries, libraries, archives, and museums) [1, 2, 3]. These collections are not only culturally and historically valuable, but also potentially susceptible to various pathways of degradation [2] caused by various indoor volatile pollutants, e.g., acetic acid, emitted by cardboard and plastic packaging boxes. Paper degradation has been extensively researched, and environmental control of temperature, humidity and UV exposure has been found to be effective in slowing decay [2, 3], but these are not the only damaging factors that affect its longevity. An often overlooked but crucial aspect of preventive conservation is the use of suitable material for enclosures, such as archival boxes. Boxes have a dual function: they physically protect the paper artifacts and act as a microclimate control system that stabilizes the object's microenvironment [4]. By creating this protective microenvironment, the storage materials protect historic paper collections from environmental fluctuations and air pollutants [5]. This function is particularly valuable in scenarios where maintaining suitable environmental conditions is a challenge. However, the effectiveness of packaging materials depends not only on their structural properties but also on their chemical compatibility with the objects [6], as promoted by ISO 16 245:2023 [7] that specifies the requirements for enclosures that are made of cellulosic materials designed to protect paper and documents, ensuring suitability for long‐term storage of paper without causing degradation, and ISO 23 404:2020 that is applicable to papers and boards used for conservation and storage of cellulose‐based collections. These standards highlight the importance of selecting packaging materials that actively contribute to the preservation of historic objects by minimizing potentially harmful chemical interactions that could lead to degradation [8].

However, it remains poorly understood if the strict standards are justified from the point of view of the emissions of VOCs [6, 9], specifically because these compounds are released not only from the storage material itself but also from paper artifacts as they degrade [8, 10]. Although it has long been thought that VOCs, particularly organic acids, significantly accelerate degradation, leading to brittleness and reduced structural integrity [9], evidence is emerging that volatile acids contribute only marginally [3, 11]. However, concerns in the conservation community remain that a reduction in the degree of polymerization (DP), leading to reduced paper mechanical stability, can be caused by VOC absorption [2, 10]. Therefore, libraries and archives have been widely using the Oddy test as a tool for the selection of suitable conservation materials for storage [12]. However, to provide more realistic risk assessments, measurement of VOC emission rates (ER) from storage materials should be considered at room temperature, ensuring that the risk is modeled at the temperature of use.

The analysis of VOCs usually proceeds using sensors, chromatography, or (mass) spectrometry [13]. Common VOC sensors include photoionization detectors (PIDs), which are frequently employed due to their rapid response and broad sensitivity [14]. Metal oxide sensors (MOSs) are also used as stand‐alone devices for VOC monitoring due to their compact size, low cost, and sensitivity to various gases, although they generally suffer from higher detection limits [15, 16]. As separation techniques, gas chromatography (GC) and liquid chromatography (LC), provide the required high selectivity. IC is the method of choice for measuring trace levels of acids, such as acetic and formic acids. Mass spectrometry (MS) detection is standard in combination with GC, providing high sensitivity [17].

Monitoring of VOCs is often based on sampling with a sorbent material, and the results of this approach depend on sorbent type and extraction method [18]. Common sampling techniques include solid‐phase microextraction (SPME) and thermal desorption sorbent tubes, e.g., Tenax TA, which can be used in both passive and active modes [17, 19]. Tenax TA sorbent tubes effectively preconcentrate VOCs, allowing for detailed analysis using GC–MS [20, 21]. SPME is also a solvent‐free method, making it suitable for applications in heritage conservation [22], however, thermal desorption tubes are more amenable to quantitation.

The aim of this study is to provide a detailed assessment of VOC emissions from a range of packaging box materials sourced from different suppliers. The study aims to quantify and characterize these emissions to determine their potential contribution to the degradation of cellulose‐based artifacts and to model the associated risk at room temperature.

## 2 Materials and Methods

Fifteen archival packaging materials from different sources were selected for this study sourced from different archival suppliers, Table 1, including ZFB (Germany), KLUG Conservation (Germany), Archival Survival (Australia), JPP (UK), and the Prague National Library (Czechia, supplier unknown), as well as an aged cardboard (supplier unknown) from the National and University Library of Slovenia (NUK) repository, presumably from the 1940s. The materials include polypropylene (PP), coated and uncoated cardboards, and specialty boards. The selection aimed to cover a diverse set of physical and chemical properties and manufacturers. The samples were tested as obtained, without any pretreatment (e.g., dehumidification or predesorption), to reflect realistic storage and use conditions.

**TABLE 1:** Description of the selected material samples.

No	Sample code	Product description	Supplier
1	PP‐ZFB	Virgin Polypropylene, hollow chamber sheets	ZFB, Germany
2	PP‐rec‐b	Polypropylene, HCS from recycled PP	ZFB, Germany
3	PP‐AS	Polypropylene	Archival Survival, Australia
4	PP‐ PNL	Polypropylene	Supplier unknown (source: Prague National Library, Czechia)
5	EB‐ZFB	Cardboard, corrugated board, EB geometry	ZFB, Germany
6	EB‐KLUG	Cardboard, corrugated board, EB geometry	KLUG conservation, Germany
7	EB‐C1	Cardboard, aqueous acrylic dispersion coating	ZFB, Germany
8	JPP	Cardboard	JPP, UK
9	JPP‐EVA	Cardboard, coated with Ethylene Vinyl Acetate (EVA)	JPP, UK
10	ResN	Paper made of natural agricultural residuals	ZFB, Germany
11	RecP	Paper, recycled from used coffee cups	ZFB, Germany
12	BagP	Bagasse paper	ZFB, Germany
13	NUK‐ref	Cardboard, naturally aged from 1940s	Supplier unknown (source: NUK, Slovenia)
14	L.138	Cardboard, ligneous corrugated (B‐flute, brown), recycled fibers	ZFB, Germany
15	L.678	Cardboard, ligneous corrugated (E‐flute, brown), recycled fibers	ZFB, Germany

The PP samples (PP‐ZFB, PP‐rec‐b, PP‐AS, and PP‐PNL) included both virgin and recycled PP, due to its widespread use in modern archival practice. Coated and uncoated cardboards (EB‐ZFB, EB‐KLUG, EB‐C1, JPP, and JPP‐EVA) were included to investigate the effects of surface treatments such as water‐based acrylic varnish and ethylene vinyl acetate (EVA) coatings.

Specialty materials, such as paper from agricultural residues (ResN), recycled paper (recP), and bagasse paper (BagP), are tested to be sustainable alternatives to conventional cardboard fiber sources. Therefore, commercial speciality papers have been provided by ZFB. In addition, aged cardboard samples (NUK‐ref) dated from the 1940s and modern lignin‐containing corrugated cardboard (L.138 and L.678), also supplied by ZFB, provided insights into the performance of old and new lignin‐containing materials.

### 2.1 Determination of pH

The pH of cellulose‐based packaging material samples was determined using the micro‐cold‐extraction method, where 0.5 mL of deionized water was added to 5.0 mg of cardboard and left overnight in a closed container.

The pH of the solution was then measured using a SevenCompact S220 pH Meter (Mettler‐Toledo, Greifensee), paired with a Micro pH combination electrode (InLab, Mettler‐Toledo, Greifensee). Calibration was performed using four buffer solutions (pH 2, pH 4, pH 7, and pH 10). The pH values of the tested materials, reported in Table 2, showed that while most samples are approximately neutral, aged cardboard (NUK) is acidic.

**TABLE 2:** pH Values of storage materials. The average standard deviation (triplicate analyses) was 0.06.

Sample	Average
EB‐ZFB	7.22
EB‐KLUG	7.01
EB‐C1	7.34
JPP	7.19
JPP‐EVA	7.34
ResN	6.43
RecP	7.09
BagP	7.26
NUK‐ref	4.33
L.138	7.14
L.678	6.91

### 2.2 Metal Oddy Test

The metal Oddy test was used to investigate the impact of the tested packaging materials on the corrosion of metals. Metal coupons were prepared from high‐purity foils supplied by Goodfellow Cambridge Ltd, Huntingdon. The metals used included silver (Ag, 99.95% purity, 0.20‐mm thickness, product code AG000420/6), copper (Cu, 99.9% purity, 0.15‐mm thickness, product code CU000650/47), and lead (Pb, 99.95% purity, 0.15‐mm thickness, product code PB000350/6). The metal coupons (measuring 35 × 10 mm) were prepared by abrading them with a micromesh cloth (grade 1800) along their length. After polishing, the coupons were immersed in a glass dish with acetone. Immediately before use, the surfaces were wiped with lint‐free tissue and dried.

Approximately 2 g of a tested material was cut into small pieces and placed in a 132‐mL glass vessel (nominal volume 100 mL, Schott, Mainz; retrace code: 00797355) [23]. The vessel also contained 0.9 mL of MilliQ water in a small vial with a cotton wool plug to maintain a humid environment. The metal coupons were inserted in a silicone stopper with three parallel slits, so they did not touch each other. The vials were sealed and kept in a laboratory oven at 60°C for 28 days. After this period, the coupons were visually inspected for discoloration, indicating corrosion and photographed on both sides using indirect lighting, to minimize spectral reflectance from the metal coupons together with a color reference chart. According to the British Museum guidelines [24], the ratings Pass (P), Temporary (T), or Fail (F) were used to assess the suitability of the packaging materials for long‐term use.

### 2.3 Paper Oddy Test

The test involved exposing paper samples to the emitted VOCs under controlled conditions to observe potential chemical changes. According to the test procedure as described in the literature [25], a sample (500±15 mg) was placed in the oven for 14 days at 80°C in a 132‐mL glass vessel (nominal volume 100 mL, Schott) [23] with a piece of Whatman No. 1 (Cytiva Europe, Breisgau) filter paper (250±10 mg). After the test, the variation in the DP of Whatman paper was determined by viscometry, following the ISO 5351:2004 standard. The results were compared to the blank (Whatman paper processed in the same conditions but with no test sample). Each sample was tested in duplicate, and each Whatman paper was analyzed twice to determine the average DP. The intrinsic viscosity [*η*] of cellulose was determined in cupriethylenediamine (CED) solution at 25°C in accordance with ISO 5351‐1:1981 [26]. The DP was subsequently calculated using the Mark–Houwink–Sakurada equation, (Equation 1) [23].

(1) $$DP^{0.85}=1.1.\left[\eta \right]$$

### 2.4 VOC Determination with TD‐GC–MS

Tenax TA sorbent tubes were used to sample VOC emissions from a subset of the studied materials. The weight of the individual samples varied slightly, within a range of ±10 mg, and the emission data were normalized accordingly. ≈280 mg of a bulk sample was weighed and put in Micro‐Chamber/Thermal Extractor (MCTE120) (Markes International, Llantrisant), preheated overnight at high temperature. The blank was measured using an empty chamber. The tubes were used to sample for 1 hr at 60°C with a purified air flow of 150 mL min^−1^. The tubes were then placed in an autosampler (Gerstel) and desorbed at 300°C for 5 min. The injection port was kept at 250°C and operated in split mode (1:100). After collection on a cryotrap at −20°C, the analytes were injected into the GC 7890A coupled to MS 5975C (both Agilent Technologies, Santa Clara, CA) equipped with a VOCOL capillary column (Merck, Darmstadt, 60 m, ID 0.25 mm, film thickness 1.50 μm). The He flow was 1.0 mL min^−1^ during the oven temperature gradient, starting at 40°C, after 5 min increasing at 20°C/min up to 220°C, and kept at this temperature for 30 min (total analysis time: 44 min). The MS transfer line was kept at 230°C and electron ionization was used (230°C). The quadrupole was kept at 150°C. The mass spectra were recorded in the m z^−1^ range 50–550 Da.

The raw chromatograms and peak areas for TD‐GC–MS experiments are available in Supporting Information 1.

### 2.5 Organic Acid Determination with IC

In order to assess and compare the overall emissions of organic acids from archival storage materials, thermal extraction was carried out at a high temperature to enhance the release of semivolatile compounds. ≈260 mg of each sample, shredded into small pieces, was placed in a Micro‐Chamber/Thermal Extractor (MCTE120). The chambers were preheated to 120°C, and kept at the same temperature during overnight sampling (17 h) to promote the desorption of acetic and formic acid. During the sampling, a purified airflow of 50 mL min^−1^ was maintained, and the emitted compounds were captured on charcoal sorbent tubes (6 × 70 mm, 100/50 mg ‐ Zefon International, Ocala, FL).

After sampling, the glass tubes were cut and the sorbent was transferred to 15‐mL tubes, separating the small and large portion. Each sorbent segment was extracted using 2 mL of 10 mM NaOH solution and sonicated for 20 min. The extracts were filtered through 0.45 µm PTFE syringe filters and analyzed using IC by injecting 25 μL into a Dionex Integrion system (Thermo Fisher Scientific, Waltham, MA), equipped with a Dionex IonPac AS11‐HC 4 × 250 mm column and a conductivity detector. A gradient concentration of potassium hydroxide KOH in MilliQ water, at a constant flow of 1.5 mL min^−1^, was used as the mobile phase, starting at 3 mM for 6 min, then increasing to 10 mM in 7 min and to 40 mM in 3 min, and after 1 min decreasing to 3 mM in 2 min over 21 min. A 40‐mA electrochemical suppressor (Thermo Fisher Scientific, Waltham, MA), was used to minimize the impact of the mobile phase on the recorded signal.

Daily, a calibration curve was produced using solutions of NaCH_3_COO, NaCl, and NH_4_HCOO. The concentrations used were: 0.1, 0.3, 0.5, 1.0, 2.5, 5.0, 7.5, and 10.0 mg L^−1^. Twelve blanks were used to calculate the limit of detection (LoD) and limit of quantification (LoQ) for the method [27], as 3x and 10x the standard deviation added to the average signal of the blank, respectively. For acetate, the LoD was 0.13 mg L^−1^, and the LoQ was 0.30 mg L^−1^. For formate, the LoD was 0.47 mg L^−1^, and the LoQ was 0.97 mg L^−1^.

This high‐temperature thermal extraction screening method enabled the identification and comparative quantification of acid emissions from different storage materials.

The raw data (peak areas, sample weights, calibration curve data) for IC experiments are available in Supporting Information 2.

### 2.6 Determination of Acetic and Formic Acid ERs

To better reflect the realistic environmental conditions in which archival materials are typically used, separate experiments were conducted to determine the ERs of acetic and formic acids at room temperature, in contrast to the exaggerated emissions observed at 120°C. In this case, ≈2–3 g of shredded material was placed in a separate microchamber (MCTE120) and left for sampling at 25°C for 19 days. A constant airflow of 50 mL min^−1^ of dry compressed air was maintained to ensure consistency across both experiments.

Charcoal sorbent tubes were used to collect the emitted acetic and formic acids, and the extracts were analyzed by IC, following the same analytical protocol as described above [28]. The LoD and LoQ were calculated on the basis of 8 determinations of blanks. For acetic acid, the LoD was 0.14 mg L^−1^and the LoQ was 0.21 mg L^−1^, and all reported concentrations were at or above the LoQ. For formic acid, the LoD was 0.36 mg L^−1^and the LoQ was 0.49 mg L^−1^.

To quantify the acetic and formic acids emissions, the normalized mass of the acid, *m*_A_ (mg g^−1^), collected from a sample was calculated using Equation (2), where *c*_extract_ represents the concentration of the collected acids in the extraction solution (mg L^−1^), *V*_extract_ is the volume of the extract (2 mL), and *m*_sample_ is the mass of the tested material (g).

(2) $$mA=\frac{C_{\text{extract}}\cdot V_{\text{extract}}}{m_{\text{sample}}}$$

The mass‐normalized ER (mg g^−1 ^min^−1^), of acetic and formic acids was calculated directly from the normalized mass of the acid (*m*_A_) and the total sampling time of 19 days at room temperature using Equation (3).

(3) $$\begin{array}{c} \text{ER}=\frac{\mathrm{mA}}{t_{\text{sampling}}}\; \end{array}$$

This equation assumes that the determined *m*_A_ does not depend on gas flow through the microchamber cell, as established before [28], and that all the emitted acetic and formic acids are quantitatively removed from the microchamber and retained by the charcoal sampling tube. This is a reasonable assumption as no breakthrough of the collected acids was detected on the sampling tubes.

### 2.7 Modeling of Equilibrium Concentrations of Acetic and Formic Acids in Archival Boxes

With the ERs determined, the next step involved the calculation of the equilibrium concentration of acetic and formic acid, *c*_eq_, within the volume of an archival box. A 2‐part box with dimensions of 21 × 29.7 × 7.4 cm was used, corresponding to a volume (***V***_box_) of 4.61 L as used in our previous research [4, 29]. The calculation of the equilibrium concentration, *c*_eq_ (µg m^−3^) (Equation 4), is based on the emission rate of the acid, ER (mg g^−1 ^min^−1^), and the air exchange rate of the box, AER (h^−1^) determined in our previous research [4].

(4) $$c_{eq}=\frac{1}{2}\cdot \frac{ER\cdot m_{\mathrm{box}}}{AER\cdot V_{\mathrm{box}}}$$

The mass of a box, ***m***_box_ (g), takes into account the 2‐part box construction. In the calculation of *c*_eq_, it can further be assumed that if a box is uncoated and stands freely on a shelf, up to half of the acids are emitted into the storage environment, however, if the boxes are stacked or if their surfaces are glued or coated, the AER can decrease to values similar to nonperforated PP boxes, and the majority of acids are emitted into the box. As AERs vary depending on the box design and construction, as well as cardboard thickness, all the calculations were made based on minimum and maximum values of AER per material type [4, 29] as per Table 3.

**TABLE 3:** Minimum and maximum AER for free‐standing and stacked boxes made of coated and uncoated cardboard and PP, as reported in [4].

	**AER (h** ^**−1**^ **)**
**Cardboard boxes**	
Min	2.65
Max	10.82
**Coated cardboard boxes**	
Min	0.46
Max	2.57
**PP boxes**	
Min	0.31
Max	0.84
Perforated PP (single sample)	6.12

## 3 Results and Discussion

A range of analytical techniques was used, operated at tailored temperature conditions based on the specific requirements and objectives for each analytical method. For the paper Oddy tests, the temperature of 80°C is normally used to accelerate VOC exposure within a practical timeframe [25]. The metal Oddy test uses 60°C as standard [24, 30]. Preliminary organic acid emission studies were carried out at 120°C overnight to enhance the desorption of acids in a practical timeframe. Finally, for acetic and formic acid ER determination, sampling was conducted at room temperature to enable modeling reflecting realistic storage conditions [29, 31].

### 3.1 Oddy Testing

The metal Oddy test results, as shown in Table 4, provide crucial insights into the corrosive effects of VOCs emitted by the tested archival materials on sensitive metal coupons: copper (Cu), silver (Ag), and lead (Pb). The evaluation was conducted following the British Museum Oddy Testing Manual [32], classifying materials into three categories: Pass (P) for no observable corrosion, Temporary (T) for slight corrosion suitable for temporary use, and Fail (F) for strong corrosion, deeming the material unsuitable for museum environments.

**TABLE 4:** Results of the metal Oddy tests per metal. The classifications into Pass (P), Temporary (T), or Fail (F) were used as per British Museum guidelines [24].

Sample	Pb	Ag	Cu
PP‐ZFB	P	P	P
PP‐rec‐b	P	P	P
PP‐AS	P	P	P
PP‐PNL	P	P	P
EB‐ZFB	P	P	P
EB‐KLUG	T	P	P
JPP	T	P	P
EB‐C1	F	P	P
JPP‐EVA	F	P	P
ResN	T	P	P
RecP	F	P	P
BagP	T	P	P
NUK‐ref	F	P	F
L138	F	P	T
L678	F	P	T

The performance of the tested materials varied significantly (Table 4). PP samples (PP‐ZFB, PP‐rec‐b, PP‐AS and PP‐PNL) demonstrated minimal corrosive impact. EB and JPP cardboards showed limited impact with P or T grades, while the coated cardboards (EB‐C1 and JPP‐EVA) displayed adverse effects due to yellow films, white crystals, and strong corrosion on lead coupons, emphasizing the impact coatings have on VOC emissions and subsequent corrosion. Specialty papers (ResN, RecP, BagP) varied in performance. BagP showed better results with mostly P and T ratings, whereas RecP exhibited significant corrosion, including large crystals and yellow films on lead coupons, resulting in a Fail grade. Typical commercial packaging cardboards from recycled and ligneous (brown) fibers (L.138 and L.678) and the naturally aged cardboard sample (NUK‐ref) presented the most severe corrosion, particularly on lead coupons, with strong white and yellow layers, green iridescence on copper, and extensive crystal formation. These materials were rated Fail, highlighting their unsuitability for archival storage of metal.

**FIGURE 1:**
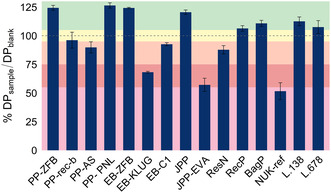
Ratio of the measured DP for the sample and the DP of the control (in %) with ranking of the effect on preservation as Preservation (>105%), Neutral (95–105%), Moderate impact (75–95%), Significant impact (55–75%), and Severe degradation (<55%) as per [25]. The error bars denote SD calculated for two repeated determinations.

In the paper Oddy tests, the DP of Whatman paper was evaluated after exposure to VOCs emitted from different materials, Figure 1. The results were compared to a protocol blank.

DP results were categorized according to their effect on preservation using the following ranking: Preservation (>105%), Neutral (95–105%), Moderate impact (75–95%), Significant impact (55–75%) and Severe degradation (<55%) [25]. The highest DP retention was recorded for EB‐ZFB, PP‐ZFB, and PP‐PNL, placing them in the preservation category and suggesting that these materials may play a stabilizing role in archiving.

L.138 and JPP also remained within the preservation range, which is interesting, given the Fail classification for L.138 in the metal Oddy test, indicating that such tests are of limited use in general risk assessment. Materials such as L.678, BagP, and RecP showed a neutral effect, indicating they are safe for use in archives. On the other hand, PP‐rec‐b, PP‐AS, ResN, and EB‐C1 fell into the moderate impact category, indicating the presence of VOC emissions that could contribute to polymer degradation over time. The most concerning results were observed for NUK‐ref, which led to the biggest DP decrease, indicating severe degradation due to VOC exposure. EB‐KLUG and JPP‐EVA also showed significant to severe degradation, indicating high VOC emissions that could accelerate the deterioration of archival paper.

In the following section, we will attempt to interpret the Oddy test results using analytical techniques, focusing on the potential sources of the observed corrosivity of the studied materials.

### 3.2 Identification of VOCs by GC–MS

**FIGURE 2:**
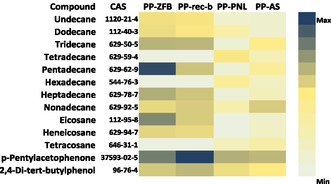
GC–MS peak areas of main VOCs detected in four polypropylene‐based storage materials, sampled using Tenax tubes.

The analysis of VOCs emitted from PP‐based archival materials using Tenax sorbent tubes coupled with GC–MS revealed a profile dominated by long‐chain alkanes (C10–C22). These hydrocarbons are typical byproducts of PP production and are known for their chemical inertness [33]. As visualized in the heatmap, Figure 2, the VOCs detected across all PP samples included not only alkanes but also minor components such as *p*‐pentylacetophenone and 2,4‐di‐tert‐butylphenol. The presence of these compounds likely reflects either residual additives or degradation products of antioxidants used in PP formulation [33, 34].

Importantly, the emission profiles were characterized by the absence of reactive or corrosive VOCs, such as carboxylic acids or aldehydes, which are commonly associated with cellulose depolymerization and metal corrosion. This benign chemical nature aligns closely with the results from the Oddy tests, in which PP samples showed minimal signs of corrosion on silver, copper, or lead coupons. Similarly, the DP results demonstrated an increase of cellulose integrity in paper samples exposed to PP materials, further confirming their stability as well as potential antioxidant properties.

**FIGURE 3:**
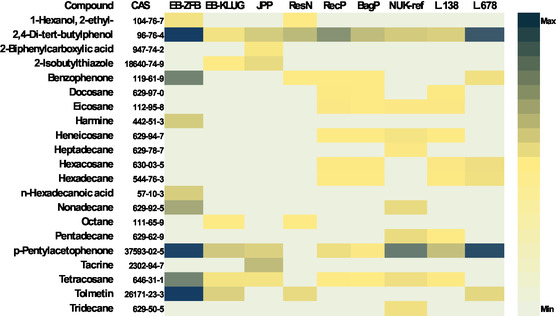
GC–MS peak areas of main VOCs detected in cellulosic‐based storage materials, sampled using Tenax tubes.

The VOC profiles of cellulosic packaging materials, visualized in Figure 3, clearly differ from those of PP‐based materials. While the latter primarily emitted inert hydrocarbons, cellulosic materials, particularly the ligneous recycling cardboard, old cardboard, and recycled paper, exhibited more chemically diverse VOC profiles, including elevated levels of long‐chain alkanes, substituted phenols, and aromatic compounds, which may reflect a history of oxidative degradation, resin, additives or processing residues [35, 36, 37].

The emission of such compounds aligns with their negative performance in the Oddy tests, where corrosion of lead coupons was observed, and is further supported by a reduction in DP of paper samples exposed to these materials, indicating possible exacerbation by emitted acids and aldehydes not typically observed in PP samples [35]. The presence of nonpolar hydrocarbons alone may have a limited impact on paper. Phenolic antioxidants could have an impact on long‐term paper stability [8, 38, 39]; however, since it was shown that acetic acid is of the biggest potential concern in paper Oddy tests [4], the rest of our research focussed on this acid.

### 3.3 Organic Acid Emissions

In addition to Tenax‐GC–MS analysis, which provides a broad profile of VOCs, targeted quantification of low‐molecular‐weight organic acids, in particular, acetic and formic acid, was performed using active sampling at 120°C, followed by IC. These acids are highly polar and often underrepresented in Tenax‐based VOC screenings due to the limited affinity of the sorbent for such small, acidic compounds.

This dual approach, a broad VOC profile and quantification of organic acid emissions, allowed for a more complete assessment of the tested materials and helped to distinguish those that emit a wide range of VOCs but pose a lower risk, and those that release smaller amounts of highly reactive compounds with greater potential for damage [31, 40, 41].

**FIGURE 4:**
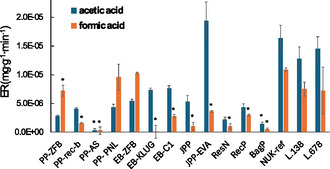
Comparison of mass‐normalized emission rates (ER) of acetic and formic acid from different packaging materials measured at 120°C over a 17‐h sampling period. The error bars denote SD calculated for two repeated determinations and the asterisks denote values below LoQ.

The analysis of acetic and formic acid emissions at 120°C revealed substantial variability among the tested materials (Figure 4). NUK‐ref and JPP‐EVA exhibited the highest acetic acid emissions. Lignin‐containing materials such as L.138 and L.678 also demonstrated higher acetic acid release, while EB‐ZFB, EB‐KLUG, EB‐C1, JPP, and RecP displayed more moderate emissions, suggesting a lower potential impact. Conversely, PP‐ZFB, PP‐PNL, PP‐AS, ResN, and BagP exhibited minimal acetic acid emissions.

Formic acid emissions followed a comparable trend, with old cardboard recording the highest emissions. PP‐PNL, EB‐ZFB, L138, L678, and PP‐ZFB also demonstrated notable formic acid release, while EB‐C1, JPP‐EVA, and recP displayed moderate to minimum emissions, suggesting a lower impact. Conversely, EB‐KLUG, JPP, PP‐rec‐b, ResN, and BagP exhibited the lowest formic acid emissions. As two observed acetic acid and 10 formic acid concentrations are below the LoQs, the data should only be interpreted in terms of trends.

However, the presence of acetic acid and formic acid emissions does not inherently render a material unsuitable for archival use. According to PAS198:2012 guideline, an acetic acid concentration of up to 100 ppb in archival environments is considered acceptable for long‐term storage [3]. Thus, materials with minor emissions may still be viable for archival storage. The next section focusses on the evaluation, whether the above observed emissions from packaging materials could lead to accumulation of acids above the suggested safe thresholds.

A limitation of this study is that relative humidity during VOC emission testing was not controlled. The microchamber system used in the study uses dry compressed air, and its humidification would have led to challenges with condensation. However, literature studies show that relative humidity typically has a smaller influence on VOC emissions compared to temperature, which directly governs ERs through volatility‐driven mechanisms [42, 43]. Relative humidity may influence VOC sorption if water is absorbed into the material more strongly than the VOC itself; however, this effect depends on both the volatile compound and the material. In contrast, temperature has been shown to exert a more significant effect on VOC emissions by increasing vapor pressure and diffusivity [44, 45]. We thus believe that the limitation is small, although this may need to be confirmed in a future study.

### 3.4 Assessment of Suitability Based on Archival Box Equilibrium Acetic and Formic Acids Content

It is clear that neither Oddy testing nor semiquantitative assessment of emissions using Tenax tubes, nor quantitative ERs at elevated temperatures, provides us with clear evidence that could support evidence‐based decision‐making at room temperature. In this section, we thus propose a new method of risk assessment, based on ER determination at room temperature followed by modeling of acetic and formic acid concentrations in an archival box. If such a concentration approaches or is higher than the threshold suggested by PAS198:2012, the material or the box design is evidently not suitable for long‐term archival storage.

To support such assessment, acetic and formic acid ERs were first assessed at room temperature (25°C). Due to the low ERs, all samples were exposed for a fixed duration of 19 days. The amount of the obtained acids through the analysis of the absorption tubes was used to calculate the mass‐normalized ER (mg g^−1 ^min^−1^) of both acetic and formic acids, calculated according to (Equation 3). The values determined in this research compare reasonably well with the value of ca. 3·10^−6 ^mg g^−1 ^min^−1^ previously reported in the literature [46] for unaged contemporary groundwood pulp paper. However, Ramalho et al. determined surface emissions, and our work reports bulk emissions, so the values are not directly comparable.

Based on the weight of a typical archival single‐walled 2‐part box (base and lid detached), calculated on the basis of box dimensions and surface weight of the packaging material, as well as on the minimum and maximum air exchange rates as determined in [4] for boxes of three types: (i) cardboard; (ii) PP; (iii) coated cardboard, it was possible to calculate the equilibrium concentration of acetic and formic acids, *c*_eq_, using (Equation 4).

For enclosure materials showing low permeability to water on one side only, such as coated cardboard [4], we can assume that the permeability to acids is lower or similar compared to water, given the sizes of molecules, leading to the conclusion that the majority of acid is emitted into the interior of the box due to the barrier. In such cases, the coefficient ½ in (Equation 4) is assumed not to be applicable.

**FIGURE 5:**
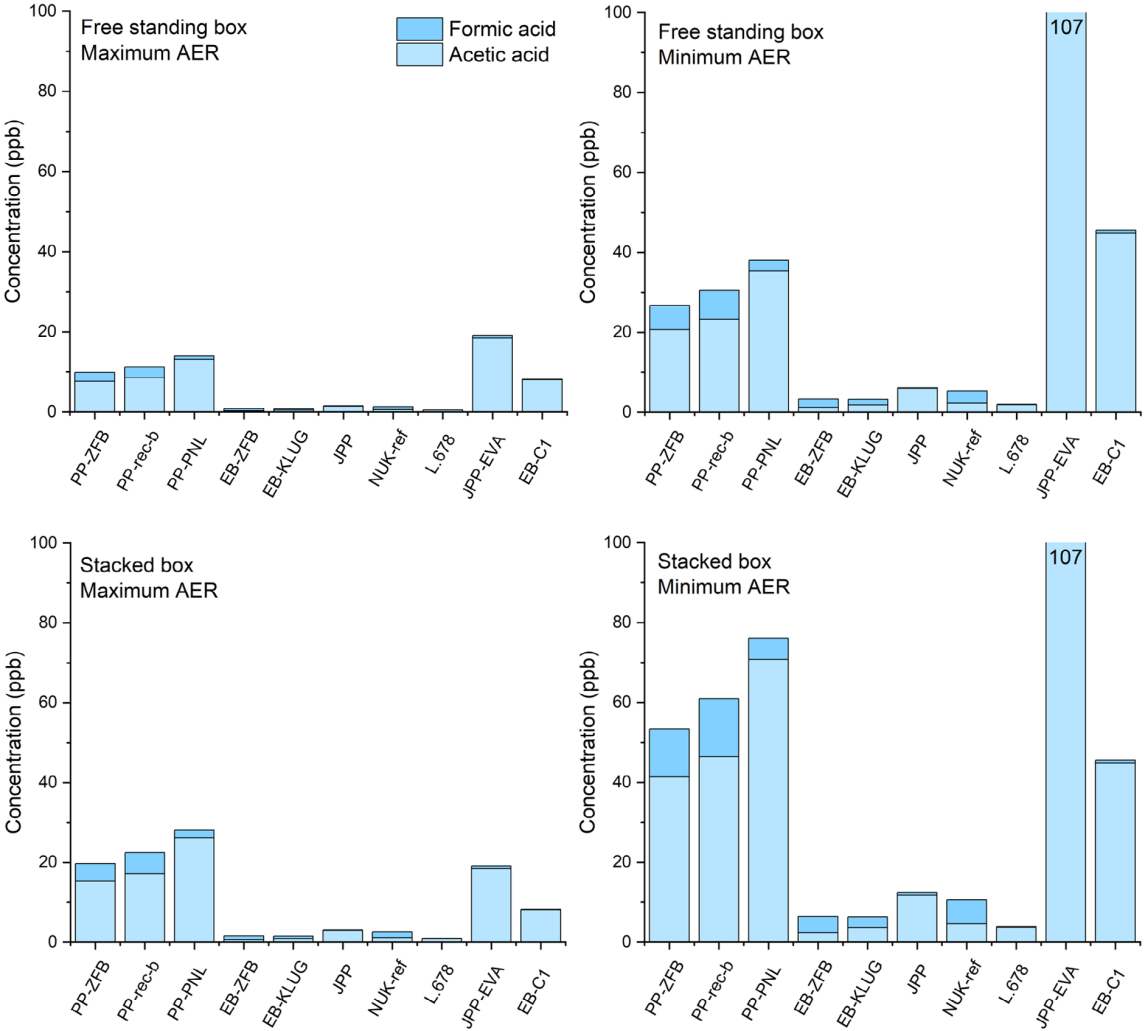
Sum of concentrations of formic acid and acetic acid, as modeled for free‐standing and stacked boxes for maximum and minimum AER scenarios, i.e., minimum and maximum concentrations, respectively. Typical ER RSD determined for PP‐ZFB and NUK‐ref was 20–30%.

This can also be assumed to be the case for boxes that are stacked, meaning that the largest sides of a box become impermeable to acetic acid. Coated cardboards that are often in use, especially in archival boxes coated with a layer of glue and decorative paper or cloth (e.g., solander boxes), also present very effectively reduced AER values that are comparable to coated cardboards [4]. Since AER also depends on box construction, as detailed in Table 3 **,** the minimum and maximum AER values sourced from [4] were used to reflect the worst‐case and best‐case scenarios, respectively, for estimating equilibrium acetic and formic acid concentrations shown in Figure 5.

Calculations of equilibrium concentrations of formic and acetic acids for free‐standing and stacked boxes, based on minimum and maximum AER scenarios, are provided in Supporting Information 3.

Across the tested materials, significant variation in *c*_eq_ was observed (Figure 5). PP‐based materials exhibited higher predicted equilibrium volatile acid concentrations compared to cellulose‐based materials. The primary reason for this lies in the low AER of PP‐based boxes, which allows the emitted acids to accumulate more readily due to limited ventilation. However, if perforated PP is used (AER 6.84 h^−1^), the predicted acid concentrations decrease to values comparable to cardboard boxes, i.e., well below 100 ppb, considered to be the safe threshold for long‐term storage according to PAS198:2012. Higher levels of accumulation can also be expected for boxes made of cardboard, where the box surface has been sealed with a layer of coating, where the AER values can decrease to as low as 0.46 h^−1^. In such cases, depending on the coating, the values may exceed the recommended threshold. It was to be expected that the EVA coating releases appreciable amounts of acetic acid, while the acrylic‐based one does not. The coating material thus requires careful consideration before application in conservation. It is worth noting that while low AER of coated cardboard boxes contributes to the accumulation of volatile acids, it also contributes to better dampening of RH within the box during periods of rapid RH change in a repository [4], and thus has particular conservation benefits.

Conversely, cardboard‐based materials consistently present lower *c*_eq_ values due to their higher AERs and lower ERs. The results demonstrate that the equilibrium concentration is not solely a function of how much acid is emitted but also depends on the box AER, which is, in turn, affected by the box construction and arrangement on a shelf (stacking).

An important conclusion of this assessment is that none of the cardboard‐based packaging materials (except EVA‐coated cardboard), regardless of the box type and regardless of age and lignin content, are likely to accumulate acetic acid in concentrations close to what PAS198:2012 would consider as the long‐term threshold concentration. For PP boxes, sufficient AER needs to be ensured.

This conclusion indicates that the lifetime of a box cannot be defined as a function of the chemical deterioration of the box material, resulting in higher acid emissions but rather as a function of the mechanical stability of a box. To assess this, however, we would need an accelerated degradation study of the cardboard, followed by mechanical testing.

The question may arise as to why concentrations of up to 70 ppb of acetic acid have been measured in archival boxes in past research [38]. If NUK‐ref weight is increased to include the emissions of material stored within, e.g., ***m***_box_ is increased tenfold, the modeled concentration of acetic acid within the box also increases tenfold and thus approaches the threshold value.

This thought experiment shows that in the case of early 20th‐century lignin‐containing archival material, we can expect elevated concentrations of acetic acid in a box; however, this is clearly not due to the packaging material, but due to the stored paper itself. An effective prevention strategy that is available would be to reduce the content mass, i.e., the amount of such paper in a box, to below 1–2 kg.

**FIGURE 6:**
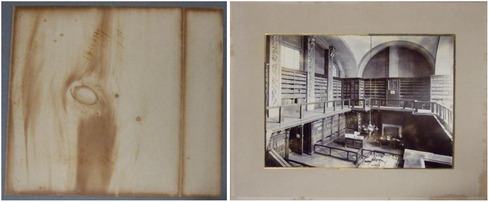
The effect of, presumably, transfer of the degradation products from a wooden backing board into the verso of a framed photograph. Left: verso of the photograph, right: recto. Photo credit: M. Strlič.

A further valid concern related to boxes made of lignin‐containing cardboard or aged cardboard could be related to the migration of degradation products, which is sometimes observed on adjacent surfaces in long‐term close contact, Figure 6, e.g., newsprint clips inserted into books. Our research shows that it is not reasonable to assume that such phenomena are due to VOC emissions. A possible alternative hypothesis is that such effects are due to the migration of degradation products. Therefore, if lignin‐containing boxes cannot be replaced in a heritage institution due to financial constraints, a lining (allowed by ISO 16 245:2023) made of an alkaline print paper containing 20% CaCO_3_ should suffice to prevent such degradation product transfer.

## 4 Conclusions

This study takes a closer look at the potential impact of VOC emissions from archival storage materials on the preservation of historic paper collections, which has in recent years become a significant conservation concern. This is often supported by Oddy tests, which in this research demonstrate diverse risks, potentially related to metal storage (metal Oddy tests). Paper Oddy tests occasionally give contradictory results to metal Oddy tests and demonstrate that VOC emissions have different effects on metals and on paper. The most questionable materials, according to the paper Oddy test, are the naturally aged, ligneous and surface‐treated (coated) cardboard materials, which also exhibit higher acetic acid and formic acid emissions at 120°C. In contrast, PP‐based materials typically show smaller acid emissions, indicating their potential safety.

To study the correlation between Oddy tests and VOC emissions, we used Tenax sampling of the emitted VOCs, followed by GC–MS and charcoal sampling of the emitted acids, followed by IC. High acetic and formic acid emissions at 120°C were associated with a 1940s archival board (NUK‐ref) and a coated cardboard (JPP‐EVA).

Interestingly, PP‐based materials exhibited high VOC emission intensity with numerous compounds identified, but still showed a positive preservation effect according to the paper Oddy test. This indicates that the total amount of emitted VOCs is not the only determinant of degradation risk; rather, the chemical nature of the emitted compounds plays a crucial role. This finding underscores the need for further chemical characterization to distinguish between potentially harmful and benign VOCs in archival storage.

Thus, although the above studies provided valuable insights, several limitations emerged, specifically the inability to use the methodology to assess the suitability of a box at the temperature of use. Second, the above methodology does not offer a clear way for assessing the lifetime of a box, should this be defined as the time at which the acid emissions of the box material exceed the threshold level concentration of volatile acids within the box.

To enable this, we measured the ERs of acetic and formic acids of packaging materials at room temperature and used the data to calculate the equilibrium concentrations within a box. It turns out that for cardboard boxes, regardless of acid ER, the box material alone cannot lead to acetic and formic acid accumulation that could exceed recommended safe thresholds set by environmental standards. This holds for new and aged lignin‐containing cardboard alike. On the other hand, PP boxes, demonstrating low air exchange rates and higher acid ERs at room temperature, could accumulate more significant concentrations of volatile acid within a box, depending on the box design. If PP use is desirable, then microperforated PP offers a suitable design solution to increase the AER of a PP box.

Our research clearly shows that the stored material itself, rather than the box, contributes to the occasionally observed high concentrations of acetic acid within a box. This can easily be managed by reducing the amount of material stored in an enclosure, especially high‐lignin‐containing paper from between 1870 and 1970, to below 1–2 kg per enclosure.

From the conservation point of view, the suitability of an archival box should thus mainly be based on its humidity‐dampening ability and on the retention of its mechanical properties, and less so on its VOC emissions.

## Supporting Information

Additional data, such as chromatograms, calibration curves and calculations of equilibrium concentrations of formic and acetic acids, are provided as Supporting Information and can be accessed at insert link here.

## Conflicts of Interest

The authors declare no conflicts of interest.

## Supporting information

Supplementary Material

## Data Availability

The data that support the findings of this study are available in the supplementary material of this article.
