# Supplementary material for: Quality Assessment of Box Materials for Long‐Term Archival Storage: VOC Emissions Are Not a Significant Concern
Source: Chempluschem. 2025 Dec 13;91(1):e202500337. doi: 10.1002/cplu.202500337 (PMC12807557; doi:10.1002/cplu.202500337)
Supplement: Supplementary file 1 — Supplementary Material [file CPLU-91-e202500337-s001.zip › cplu.70092-sup-0002-supdata-S2.pdf]

Sampling Time (hr)	Air Volume (L)
0.25	4.5
0.5	9
1	18
3.25	58.5
24	432
48	864
75	1350
96	1728

Tube number	Tubes portions	Peak area
1	Front	0.4223
	back	0.011
2	Front	0.7716
	back	0.0131
3	Front	1.3401
	back	0.0105
4	Front	3.0778
	back	0.0103
5	Front	13.8104
	back	0.011
6	Front	17.058
	back	0.2427
7	Front	20.369
	back	3.6353
8	Front	20.3405
	back	8.7684

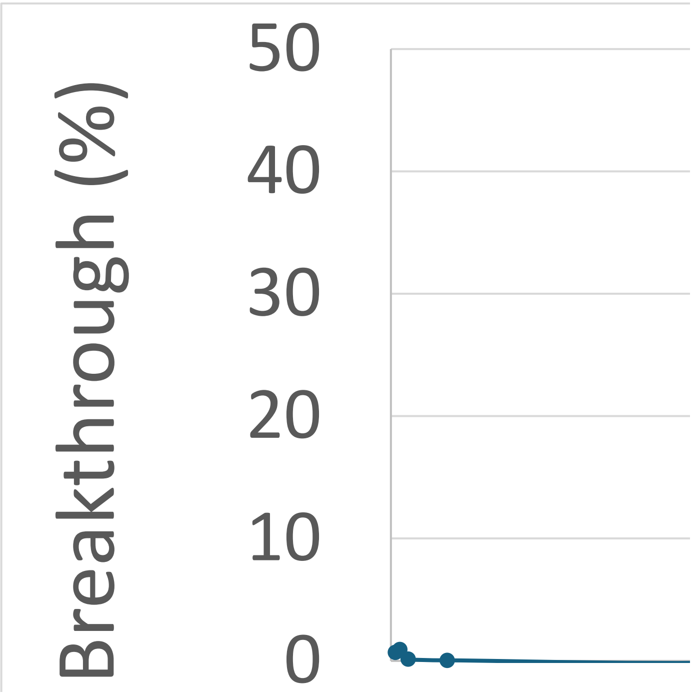

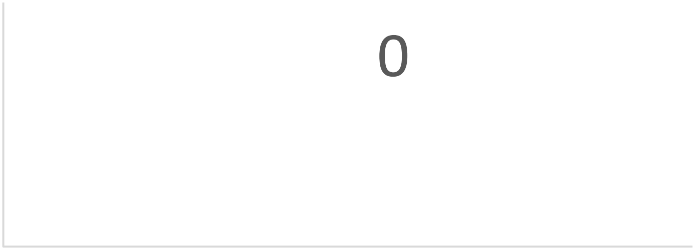
0

AA Concentration (ug/L) CONSENANT	mAA (mg)
2.9809	0.01341405
2.9809	0.0268281
2.9809	0.0536562
2.9809	0.17438265
2.9809	1.2877488
2.9809	2.5754976
2.9809	4.024215
2.9809	5.1509952

Collected concentration (mg/g)	Concentration Front+Back (mg/g)
0.13	0.13
0.00	
0.24	0.24
0.00	
0.42	0.42
0.00	
0.96	0.96
0.00	
4.34	4.34
0.00	
5.38	5.53
0.15	
6.43	8.72
2.29	
6.40	11.92
5.52	

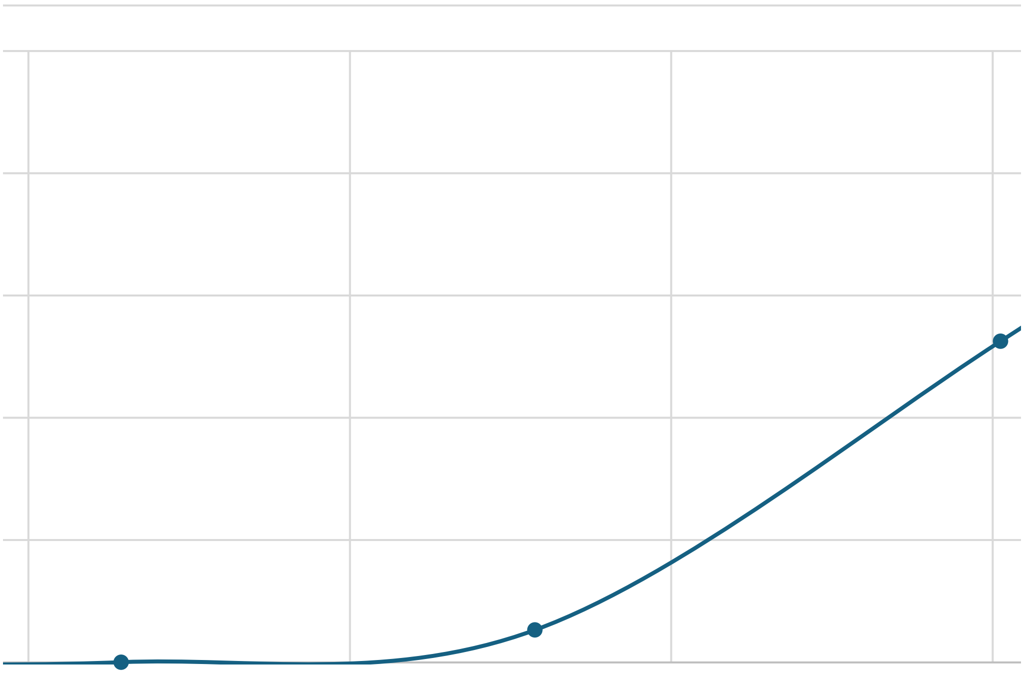

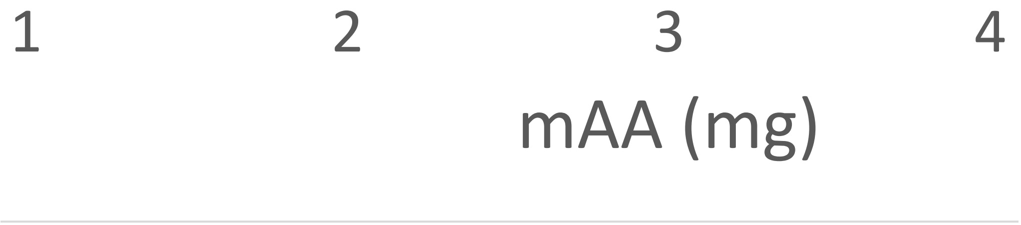
1
2
3
4
mAA (mg)

Breakthrough (%)
0.69
0.92
0.14
0.05
0.02
2.65
26.26
46.30

Breakthrough (%)
0.69
0.92
0.14
0.05
0.02
2.65
26.26
46.30

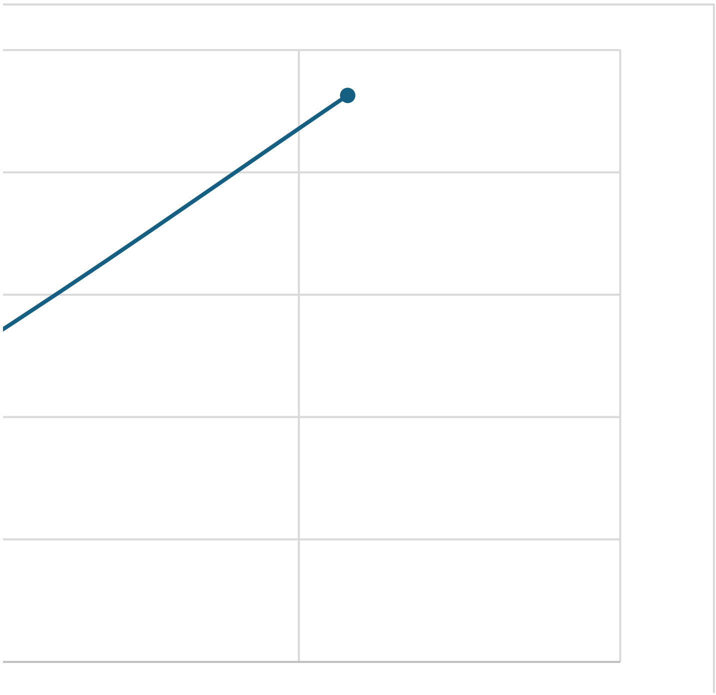

5	6
