# Supplementary material for: Quality Assessment of Box Materials for Long‐Term Archival Storage: VOC Emissions Are Not a Significant Concern
Source: Chempluschem. 2025 Dec 13;91(1):e202500337. doi: 10.1002/cplu.202500337 (PMC12807557; doi:10.1002/cplu.202500337)
Supplement: Supplementary file 1 — Supplementary Material [file CPLU-91-e202500337-s001.zip › cplu.70092-sup-0003-supdata-S3.pdf]

Material	Free-standing box	
	c_{eq} min AA	c_{eq} max AA
	(ppb)	(ppb)
PP-ZFB	7.63	20.69
PP-rec-b	8.56	23.20
PP-PNL	13.06	35.38
EB-ZFB	0.29	1.18
EB-KLUG	0.45	1.83
JPP	1.44	5.88
NUK-ref	0.56	2.29
L.678	0.46	1.87
JPP-EVA	18.40	102.82
EB-C1	8.03	44.85

		Stacked box
c_{eq} min FA	c_{eq} max FA	c_{eq} min AA
(ppb)	(ppb)	(ppb)
2.21	6.00	15.27
2.68	7.28	17.13
0.98	2.65	26.11
0.51	2.06	0.58
0.33	1.35	0.90
0.07	0.30	2.88
0.73	2.98	1.12
0.03	0.10	0.91
0.73	4.08	18.40
0.13	0.72	8.03

c_{eq} max AA	c_{eq} min FA	c_{eq} max FA
(ppb)	(ppb)	(ppb)
41.37	4.43	12.00
46.40	5.37	14.55
70.76	1.96	5.30
2.36	1.01	4.13
3.66	0.66	2.70
11.77	0.15	0.61
4.57	1.46	5.97
3.73	0.05	0.20
102.82	0.73	4.08
44.85	0.13	0.72
