# Supplementary material for: Quality Assessment of Box Materials for Long‐Term Archival Storage: VOC Emissions Are Not a Significant Concern
Source: Chempluschem. 2025 Dec 13;91(1):e202500337. doi: 10.1002/cplu.202500337 (PMC12807557; doi:10.1002/cplu.202500337)
Supplement: Supplementary file 1 — Supplementary Material [file CPLU-91-e202500337-s001.zip › cplu.70092-sup-0001-supdata-S1.pdf]

Title

Quality Assessment of Box Mate
VOC Emissions Are not a Signifi

Authors

Randa Deraz[a*], Fabiana Di Gianvincenzo[
Elnaggar[a,d], Matija Strlič[a,e]

Supplementary Information 1

Raw Gas Chromatography–Mass Spectrometry (G
corresponds to one sample and includes the full c
areas and their corresponding retention times

rials for Long-Term Archival Storage:
cant Concern

a,b], Jasna Malešič[c], Irena Kralj Cigić[a], Abdelrazek

;C-MS) data for 15 packaging material samples. Each sheet
hromatogram raw data along with a list of integrated peak
